# Interactions between Seagrass Complexity, Hydrodynamic Flow and Biomixing Alter Food Availability for Associated Filter-Feeding Organisms

**DOI:** 10.1371/journal.pone.0104949

**Published:** 2014-08-27

**Authors:** Vanessa González-Ortiz, Luis G. Egea, Rocio Jiménez-Ramos, Francisco Moreno-Marín, José L. Pérez-Lloréns, Tjeed J. Bouma, Fernando G. Brun

**Affiliations:** 1 Department of Biology, Faculty of Marine and Environmental Sciences of University of Cadiz, Puerto Real, Cadiz, Spain; 2 Department of Spatial Ecology, Netherlands Institute for Sea Research, Yerseke, The Netherlands; Dauphin Island Sea Lab, United States of America

## Abstract

Seagrass shoots interact with hydrodynamic forces and thereby a positively or negatively influence the survival of associated species. The modification of these forces indirectly alters the physical transport and flux of edible particles within seagrass meadows, which will influence the growth and survivorship of associated filter-feeding organisms. The present work contributes to gaining insight into the mechanisms controlling the availability of resources for filter feeders inhabiting seagrass canopies, both from physical (influenced by seagrass density and patchiness) and biological (regulated by filter feeder density) perspectives. A factorial experiment was conducted in a large racetrack flume, which combined changes in hydrodynamic conditions, chlorophyll *a* concentration in the water and food intake rate (FIR) in a model active filter-feeding organism (the cockle). Results showed that seagrass density and patchiness modified both hydrodynamic forces and availability of resources for filter feeders. Chlorophyll *a* water content decreased to 50% of the initial value when densities of both seagrass shoots and cockles were high. Also, filter feeder density controlled resource availability within seagrass patches, depending on its spatial position within the racetrack flume. Under high density of filter-feeding organisms, chlorophyll *a* levels were lower between patches. This suggests that the pumping activity of cockles (i.e. biomixing) is an emergent key factor affecting both resource availability and FIR for filter feeders in dense canopies. Applying our results to natural conditions, we suggest the existence of a direct correlation between habitat complexity (i.e. shoot density and degree of patchiness) and filter feeders density. Fragmented and low-density patches seem to offer both greater protection from hydrodynamic forces and higher resource availability. In denser patches, however, resources are allocated mostly within the canopy, which would benefit filter feeders if they occurred at low densities, but would be limiting when filter feeder were at high densities.

## Introduction

Seagrasses are important ecosystem engineers, which can change the physical environment through their physical structures [1]. Such habitat modification can result in positive feedbacks, stabilizing seagrass meadows [2] as well as having either a positive (e.g. facilitation) or negative effects on the survival of associated species [3], [4]. Numerous studies over the last decades have explored how physical and biological habitat modification promoted by seagrass meadows affects the occurrence of filter-feeding infauna (e.g. distribution, survival, growth, etc.), which constitutes an ecologically and economically important group of marine species [5]–[13].

In soft-bottom coastal areas, cockles have been shown to filter particles from the water column by raising their siphons up from the sediment and pumping water and food particles into their digestive system, resulting in important implications at the ecosystem level [15]–[19]. Some studies have highlighted the importance of the effects promoted by seagrass beds on the food supply, growth and survival of filter feeders [6], [10], [20]. Filter feeder activity is highly dependent on the physical transport of edible particles in the water and flow speed [21], [22]. Classical theories suggest that filter and suspension feeders should benefit from higher water refreshment rates because of simultaneously higher mixing rates and particle fluxes (i.e. food availability) [23] within the benthic boundary layer [21], [24], [25]. In contrast, it has been suggested that calmer conditions would facilitate trapping of food particles [6], [12] or increase their consumption by filter feeders, which are more stressed at high flow regimes; [21], [23], . However, other studies have pointed out in turn that the reduction of particle fluxes associated with attenuated conditions within the canopy could fully deplete resources within the bottom benthic boundary layer and thus negatively affect the growth rates of filter feeders [8], [11], [14], [30], [31].

The spatial distribution of food particle concentration (particles · ml^−1^) within a seagrass canopy will be the outcome of the balance between food input and consumption, with input depending strongly upon the volumetric flow rate within the canopy and the concentration of particles in the bulk water [32]. The degree to which seagrasses modify water flow depends largely on their own morphological and architectural complexity. From a physical perspective, dense canopies enhance both particle collision and sedimentation [33]–[37] because of the reduction in flow velocity and lost of momentum in the particles. Thus, in dense meadows with low particle flux and high competition for these resources (e.g. high filter feeder density) this could result in particle depletion (i.e. low concentration) within the canopy [38] and low survival rates [12]. In contrast, thinner canopies may allow for higher passage of water flow and constant renewing of particles across the bed, which could have potentially positive effects on the associated fauna [12], [39]. In addition to vegetation density, landscape fragmentation also strongly affects water flow because of the enhancement of the “edge effect” in patchy meadows [40]. Edges are transitional zones, where substantial changes in hydrodynamic variables (e.g. vertical turbulence) and biological processes [14], [32], [41] co-occur, affecting distribution patterns of edible particles throughout the seagrass bed [42], [43]. Although consumption rate mostly depends on the feeding activity [44], the effect of seagrass presence on filter feeder activity remains unclear, although it has been shown to reduce predation on filter feeders [6]. This uncertainty may be due to the complexity of the overall effect, with possibly contrasting effects depending on the interaction of *i)* the flow rate within the meadow in relation to its structural characteristics (density and patchiness) and *ii)* the number of filter feeders in combination with their levels of activity in filter-feeding. Thus, the link between physical interactions (plant structure-water flow influenced by the complexity of the seagrass meadow) and biological ones (consumption and bio-mixing) will play a crucial role in determining the distribution and composition of the filter feeder community.

To our knowledge, none of the published studies on the effects of the presence of seagrasses on filter feeders used a full factorial design to explore the relationship between the physical effect (promoted by seagrasses) and the biological one (fostered by the faunal bio-mixing and consumption of food resources) on the food intake rate (FIR, µg Chla·l^−1^·h^−1^) of an active filter feeder. The present work contributes to gaining insights into the biological and physical mechanisms controlling food supply to filter feeders inhabiting seagrass canopies. Therefore, the specific objectives of our study were: (1) to establish the relationship between hydrodynamics (flow and turbulence) and seagrass complexity (shoot density and number of patches as a proxy for landscape patchiness), (2) to measure how variations in flow characteristics alter the availability of resources for filter feeders (within and outside the meadows), (3) to estimate if flow and food concentration were modified by the presence of high densities of an active filter feeder (i.e. the cockle, *Cerastoderma edule* Linnaeus, 1758), and (4) to check whether the interaction of both seagrass complexity (i.e. a proxy for physical effect) and number of filter feeding organisms (i.e. a proxy for biological effect) could alter the FIR. To achieve these goals, a racetrack flume experiment combining different seagrass shoot densities (low and high) and degree of patchiness (1 patch and 2 patches) with the absence and presence (at high densities) of a filter feeder, the cockle, was carried out at the Netherlands Institute of Sea Research.

## Materials and Methods

### Artificial seagrass (mimics) design

**Figure 1:**
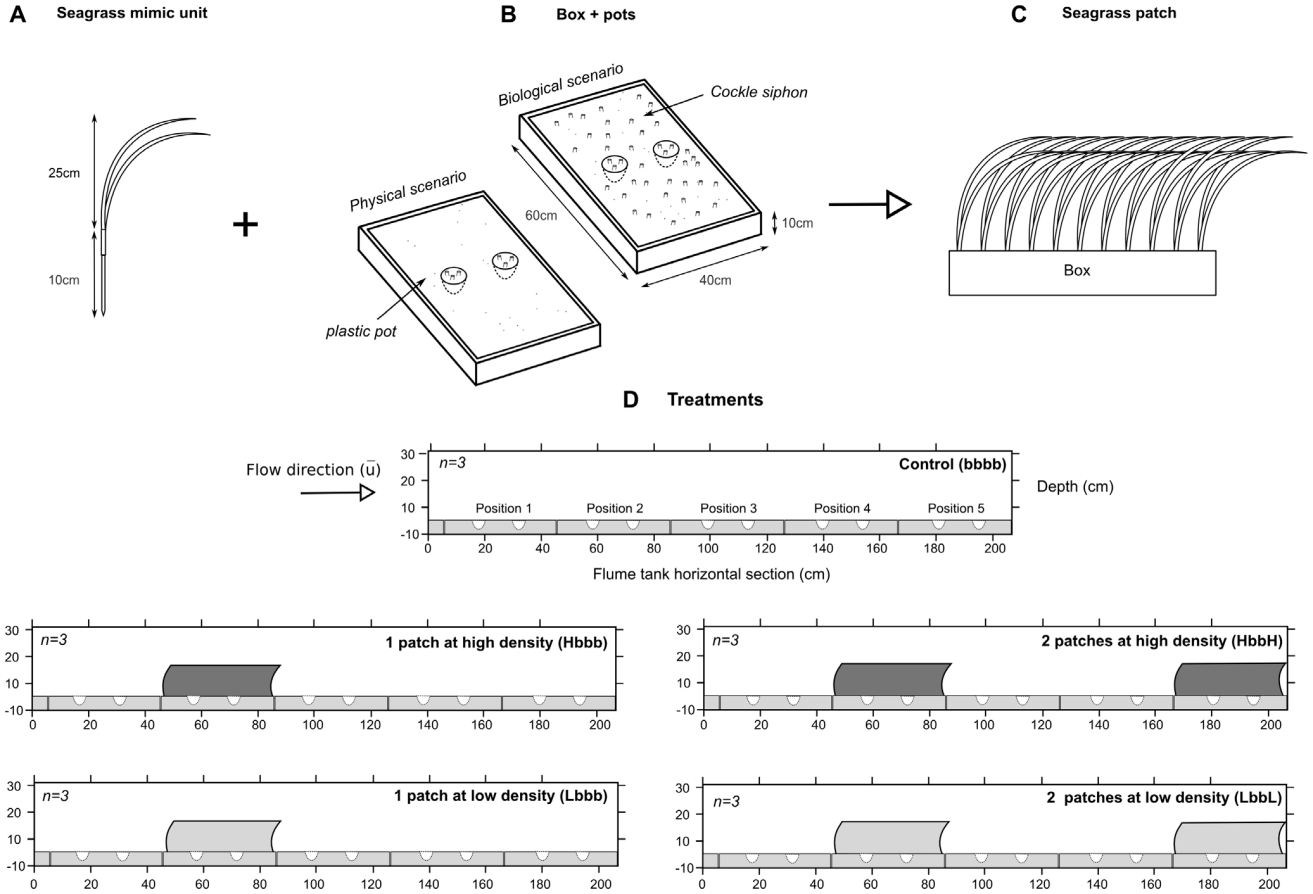
Drawing of the experimental set-up with views shown across the racetrack flume channel.

Seagrass mimics were designed to simulate the main physical properties of the submerged vegetation of coastal areas. Manipulating the different treatments involving changes in architectural characteristics of the bed (e.g. leaf length, shoot density, patch size, etc.) was largely facilitated by the use of mimics [45], [46]. Mimic structure was as follows: above-ground shoots were simulated by using a group of leaf-like plastic straps, which were attached to a wooden stick, simulating the rhizome-root system [47], through a 4×0.4 cm plastic straw filled with adhesive silicon (imitating the leaf sheath) (Figure **1**A). Morphometric characteristics of the mimicked leaves (length, width and thickness; see Figure **1**B) resembled those of the main species thriving in European Atlantic coasts, *Zostera noltei*, *Z. marina and Cymodocea nodosa*. The wooden stick kept the plastic straps anchored into the sediment, somewhat mimicking the belowground function of real vegetation. This design ensured both buoyancy and sediment permeability through the belowground structures for the filter feeders avoiding any kind of biological interaction plant-animal (i.e. grazing, herbivory or chemical interactions).

### Racetrack flume set-up

Experiments were run in a large unidirectional racetrack flume tank ([45], see Figure **1**) with a test section of 200×60 cm and a total length of 1700 cm. Nine wooden boxes (60×40×10 cm^3^) were constructed to create a kind of “seagrass puzzle system”, where boxes (i.e. the puzzle pieces) with and without mimics could be placed interchangeably with each other at several positions (5 positions in total) within the tank to facilitate the run of the different treatments (Figure **1**B). Two small plastic pots were fixed at the center of each box, then boxes were then filled with muddy sediment and mimics were planted inside (Figure **1**C) using the following scheme: (1) two boxes representing low-density patches (L, 500 shoots m^−2^); (2) two boxes representing high-density patches (H, 1500 shoots m^−2^); and (3) five boxes without mimics to represent the bare spaces between patches (b). The flume tank was filled with natural seawater (water column height of 0.4 m) and a bare box (b) was always placed 40 cm in front of the test section (position 1). Thereafter, four of the aforementioned boxes were placed into the test section according to the following treatment combinations (treatments): (1) one L box (position 2) followed by three b boxes (positions 3 to 5) (Lbbb-treatment); (2) two L boxes (positions 2 and 5) separated by two b boxes (positions 3 and 4) (LbbL-treatment); (3) one H box (position 2) followed by three b boxes (positions 3 to 5) (Hbbb-treatment); (4) two H boxes (positions 2 and 5) separated by two b boxes (positions 3 and 4) (HbbH-treatment); and finally a control treatment with four b boxes (positions 2 to 5) (bbbb-control treatment) (full details in Figure **1** and **Table 1**). All the treatments were done in triplicate (n = 3).

**Table 1:** Glossary: Summary table with the description of the most important terms used in this work.

Term	Definition	Equivalence
Pot	*Small plastic container hosting 3 experimental cockles.*	*1 Pot = 3 cockles*
Box	*Square wooden container (60×40×10 cm), filled with sediment, where two pots (6 cockles in total) were placed. Five boxes in total were used per experimental trial. In two of them, seagrass mimics were planted at low shoot density (L), while in the other two, they were planted at high shoot density (H). The rest were filled only with sediment (b)*	*1 box = 2 pots = 6 cockles*
Position	*Location of the boxes along the racetrack flume in a set order of 1–5, where box 1 was at the foreground flow direction (left) and box 5 at the downstream rearmost position (right). Details in * *Fig. 1* *.*	*Position = numbered box (box 1 to box 5) = 6 cockles.*
Shoot density	*Number of mimics (shoots) per square meter. Some boxes simulated low-density patches (L, 500 shoots m^−2^), others high-density patches (H, 1,500 shoots m^−2^) and also some boxes simulated bare sediment (b, 0 shoots m^−2^).*	*L = box with 500 shoots m^−2^. H = box with 1,500 shoots m^−2^.*
Number of patches	*Number of boxes with mimics. Two levels were used in the treatments: one box (one patch; e.g. Lbbb) or 2 boxes (two patches; e.g. LbbL).*	*One patch = 1 box with mimics in position 2. Two patches = 2 boxes with mimics in positions 2 and 5.*
Physical scenario	*Experimental approach where physical structures of mimics were the main factor responsible for the changes in food availability within the canopy (e.g. changes in volumetric flow rate or turbulence).*	*Physical scenario = very low density of filter feeders.*
Biological scenario	*Experimental approach where physical structures of mimics plus the presence of high densities of filter feeders were the main factors responsible for the changes in food availability within the canopy (e.g. changes in volumetric flow rate, turbulence, biomixing and consumption).*	*Biological scenario = high density of filter feeders.*
Food availability	*Food particles available over a time interval (particles· cm^−1.^s^−1^) controlled by the velocity of the flow and the complexity (i.e. number of structures) of the seagrass bed.*	
Food concentration	*Food particles in the water (particles·ml^−1^) accumulated within the canopy, dependent on the balance between inputs (deposition) and consumption by filter feeders.*	

**Figure 2:**
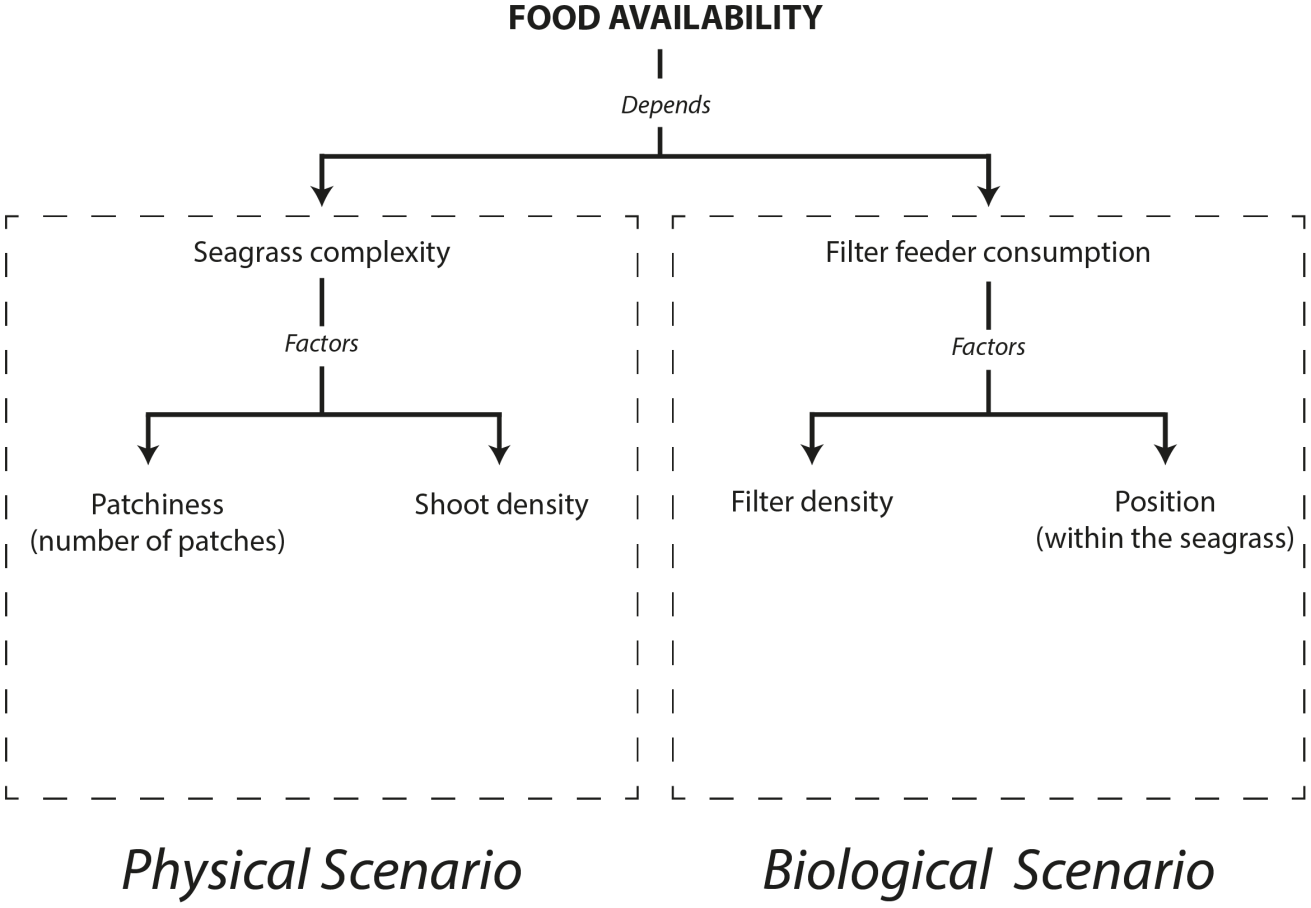
Tree diagram showing the relationships among all the measured factors.

To discriminate between physical (reduction of volumetric flow rate) and biological (filtering activity of organisms) factors controlling the resource availability and concentration for the filter feeders, treatments were carried out at two contrasting densities of filter feeders (Figure **2**). The very low density treatment of filter feeders was referred as the “physical scenario”, since density was too low to explain shortages in food supply, whereas the high density treatment was called the “biological scenario” (see below).

### Cockles sampling

The filter feeder cockle (*Cerastoderma edule*) was chosen due to its abundance and reliable behavior as model organism [12], [48], [49]. Adults were collected by hand from a muddy shore at KrabbenKreek (51° 37′ 17″N, 4° 6′ 59″E, Oosterschelde Estuary, the Netherlands) during low tide and transported within one hour to the laboratory. As Oosterschelde Estuary is a Natura 2000 reserve, permission for scientific activities was obtained from the province of Zeeland and the “stichting Het Zeeuwse Landschap”. Cockles had on average a shell length of 27.21±0.54 mm and a fresh weight of 1.68±0.09 g FW (only soft tissues, without the shell). Individuals were acclimatized to experimental conditions for a period of 7 days in a large reservoir with flowing water and constant temperature (21°C). A culture of *Isochrysis galbana* Parke, 1949 was used as food source during the period. Before any experimental run, some cockles (thereafter called “experimental cockles”) were randomly selected from this large reservoir and starved for 24 hours in a 300 L oxygenated tank with circulating seawater. In both scenarios (physical and biological) 3 “experimental cockles” each placed in both small plastic pots at the center of each box (3×2 = 6 cockles per position) and gently buried. This was done in all boxes from those in position 1 (leading edge of the flume) up to those in position 5 (downstream in the flume). In the biological scenario some starved cockles (30 per box) in addition to the “experimental cockles” were haphazardly distributed over all boxes to reach densities of 133 cockles·m^−2^ (recorded natural density in the area [50], see Figure **1**A for more details). In addition, 10 starved cockles were frozen before each run to estimate the initial chlorophyll stomach content.

### Experimental set-up

A unidirectional flow velocity of 15 cm·s^−1^ was chosen according to values measured at the cockle sampling site (from 5 cm·s^−1^ to 25 cm·s^−1^) [45]. Before starting each of the runs, the flow velocity of the racetrack flume was left to stabilize for 15 min, to the flow conditions, and an aliquot (50 mL) of concentrated *Isochrysis galbana* culture was added to the flume close to the drive belt to achieve an initial chlorophyll *a* concentration in the water of approximately 3.5±1.5 µg Chl *a* L^−1^. Regardless of the experimental treatment, the racetrack flume was run for 1 hour in each trial. Once the experimental period ended, “experimental cockles” were removed from pots and immediately frozen at −20°C for further analysis. Subsequently, hydrodynamic characterization was done in all the trials (see below). In the biological scenario, the additional cockles added into the boxes were also removed and returned to the acclimation reservoir.

### Water chlorophyll measurements in the racetrack flume

**Figure 3:**
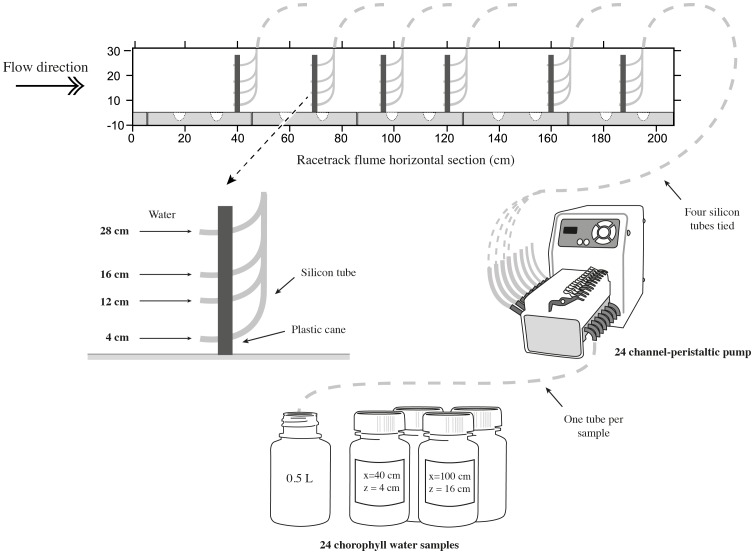
Detailed drawing of the chlorophyll sampling set-up.

Three water samples (1 L per sample) were taken: i) one before adding the algal culture into the racetrack flume (Chla_flum_), ii) one 10 min following the algal culture addition to estimate the initial chlorophyll *a* concentration at the beginning of each run (Chla_init_), and iii) one additional water sample once the experiment ended to check the final chlorophyll *a* concentration in the flume (Chla_fin_). Furthermore, to detect likely chlorophyll *a* gradients in the water (both *x* and *z* axis) resulting from cockle feeding activity, a set of silicon tubes simulating cockle siphons were attached at different heights (*z* = 4, 12, 16 and 28 cm from the bottom) of a plastic cane [51]. Then, several sets were placed along the test section of the racetrack flume (*x* = 40, 70, 100, 120, 160 and 185 cm) in the centre of the channel (*y* = 30 cm) and connected to a 24 channel-peristaltic pump (Watson Marlow 205s) (Figure **3**). Water sampling (24 samples per set, approximately 0.5 L per sample) started 40 minutes after the experiment began and lasted for 20 min. Water samples were immediately filtered at low vacuum (Whatman GF/F, 0.7 µm filters), stored in labeled aluminum envelopes and frozen at −80°C until chlorophyll *a* analysis. Chlorophyll *a* was extracted by soaking the samples in acetone (90%) in darkness (24 h at 4°C), and the supernatant was measured using spectrophotometry [52]. Mean chlorophyll *a* values (n = 3) were interpolated along the tank sections (*x* and *z* axes) as chlorophyll percentage (%) with respect to the initial chlorophyll concentration (Chla_init_) according to eq. (1). Thus, a value of 100% indicated that measured Chla concentration at the end of the experimental period exactly equaled Chla_init_ and 0% denoted the total disappearance of initial Chla.
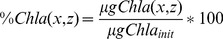
(1)


### Hydrodynamic measurements in the racetrack flume

Once an experimental period ended and “experimental cockles” were carefully collected, the flume was left running (at the same flow velocity) to characterize the hydrodynamic environment. The three components of velocity (*u* [horizontally parallel to the flume], *v* [vertical] and *w* [horizontally perpendicular to the flume]) were measured at 10 Hz with an acoustic Doppler velocimeter (Nortek ADV). The hydrodynamic variables estimated were: (1) the velocity profile 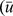, cm·s^−1^) including the vector of direction; (2) the turbulent kinetic energy (TKE, cm^−2^·s^−2^) as a proxy for the turbulent energy per mass of fluid; and (3) the Reynolds stress (τ_R_, Pa) as a proxy for the vertical transfer of turbulence. In all the treatments, a 3D grid consisting of 98 points regularly distributed in 14 steps of 0.15 m along the *x* axis (from *x* = 0 to *x* = 195 cm), 7 steps of 0.05 m along the *z* axis (from *z* = 1 in the bottom to *z* = 31 cm) and 3 points along the *y* axis (*y* = 19, *y* = 24 and *y* = 29 cm) was used. At all points the ADV was positioned for 50 s, rendering 500 measurements per point (i.e. 10 measurements per second—10 Hz—during 50 s). The hydrodynamic parameters were corrected by removing those data with correlations below 70% (low correlation indicates unreliable data) as done by Morris et al. [32]. The velocity vector and the average velocity along the *x* axis 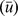, turbulent kinetic energy (TKE = 0.5·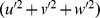) and Reynolds stress (τ_R_ = 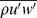) were calculated according to published equations [46], [53]. To get an overview of the hydrodynamic effects promoted by patchiness, hydrodynamic data were pooled within each of the 5 boxes along the *x*-axis (from position 1 to 5; Figure **1**D).

### Statistics analysis

Significant differences in hydrodynamic parameters (i.e. 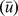, TKE and τ_R_) were checked using a 3-way fixed-factor ANOVA (number of patches, shoot density and position) separately in both scenarios (physical and biological). This method was used in order to give a simple and comprehensible framework for the interrelation of multiple factors and their effect on the FIRs of the filter feeders. Data normality and homoscedasticity were checked before the ANOVA. To test for significant differences in the cockle FIR among different treatments and positions, a non-parametric Kruskal-Wallis test was applied, since data were not normally distributed even after applying several different transformations. Data are presented as mean ± 1 standard error and significance levels were set at *p* = 0.05.

## Results

### Interaction between mimics and flow

**Figure 4:**
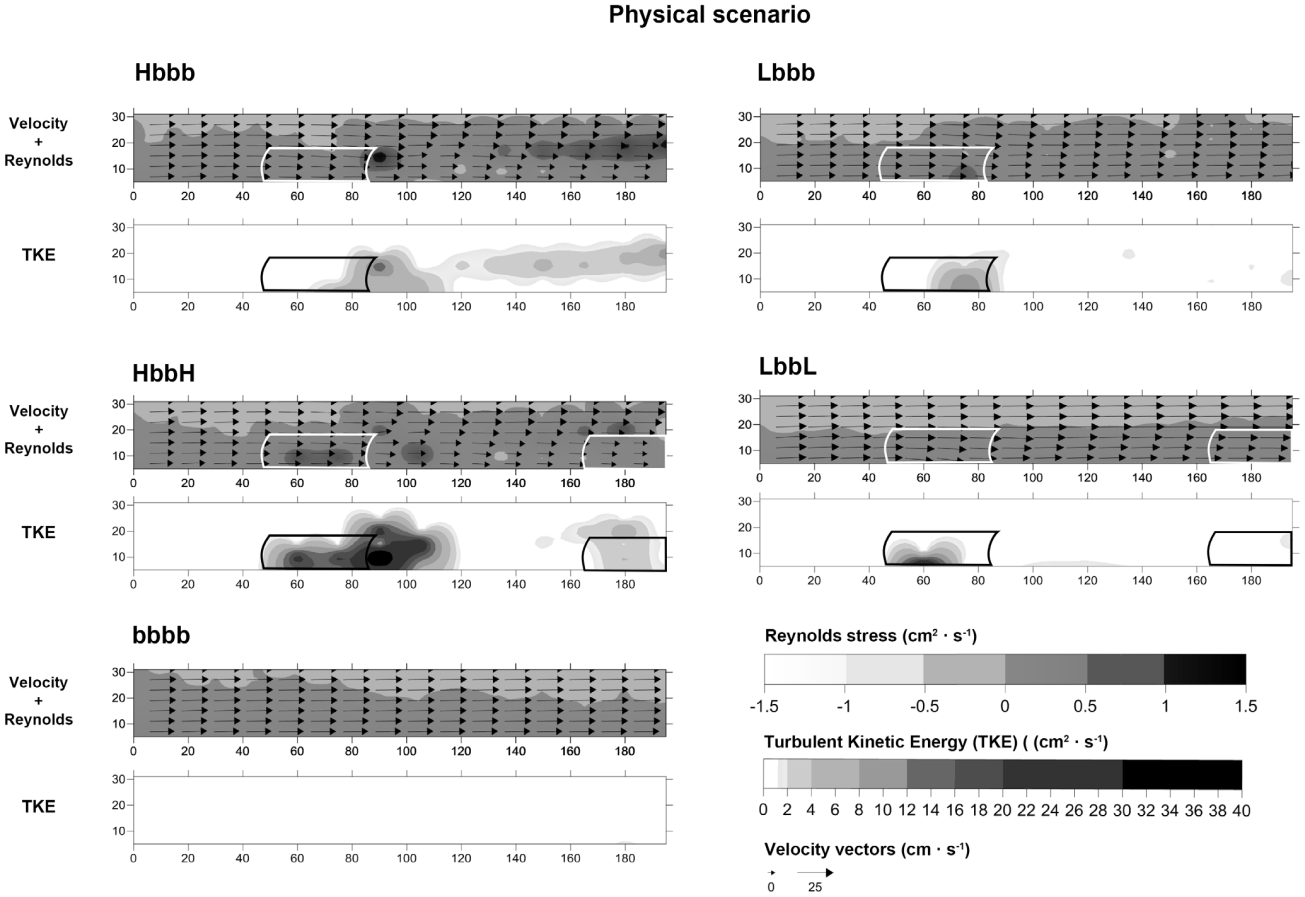
Vector plots along the horizontal axis 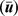 measured for different treatments in the physical scenario and Reynolds stress (τ_R_) and TKE values. The graduated grey shading outlines the extent of the patch canopies.

**Figure 5:**
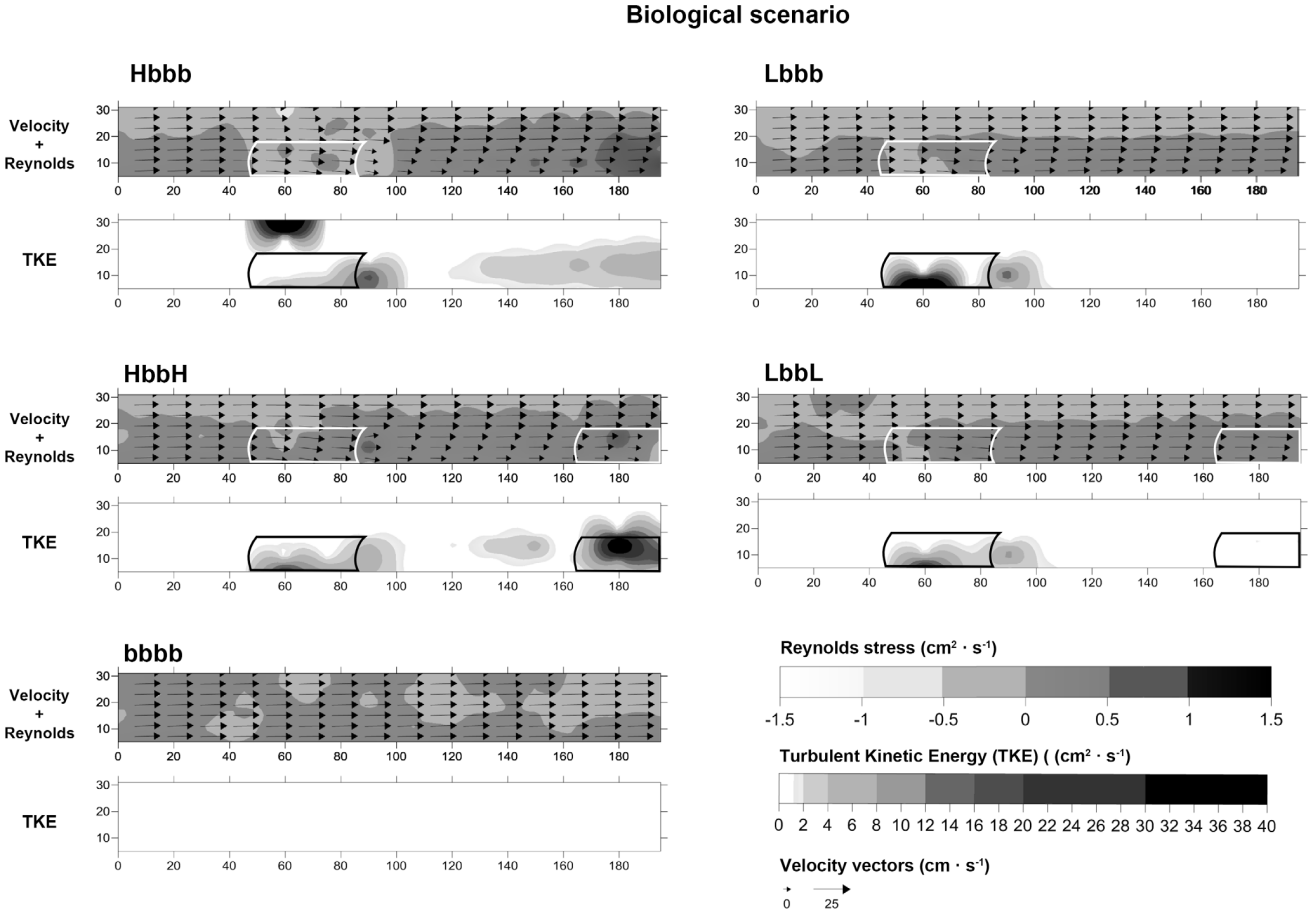
Vector plots along the horizontal axis 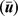, measured for different treatments in the biological scenario and Reynolds stress (τ_R_) and TKE values. The graduated grey shading outlines the extent of the patch canopies.

Flow velocity 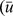, cm·s^−1^) showed a significant spatial reduction along the *x-z* plane of the racetrack flume with both increasing shoot density and with the number of patches (Hbbb and HbbH) regardless of the scenario (physical or biological, Table **2**, Figures **4** and **5**). In contrast, no differences were found in unvegetated controls (bbbb) under either biological or physical scenarios (K-W *p*-value<0.05; Table **3**). Overall, a well-formed TKE wake behind the first patch (position 3) was observed, especially in the high shoot density treatments (Hbbb and HbbH). In the physical scenario, TKE values were modified by shoot density, number of patches and position (Table **2**, Figure **4**), whereas only the interaction between shoot density and position had a significant effect in the biological scenario (Table **2**, Figure **4**). In the physical scenario, TKE values increased with shoot density and number of patches: from 0.29±0.06 cm^−2^·s^−2^ (control, bbbb) to 8.30±3.50 cm^−2^·s^−2^ (HbbH) in the first patch (position 2), and from 0.38±0.17 cm^−2^·s^−2^ (bbbb) to 1.47±0.51 cm^−2^·s^−2^ (HbbH) in the second patch (position 5). A similar increase in TKE values were also recorded in the biological scenario (from 0.12±0.06 cm^−2^·s^−2^ (bbbb) to 12.41±6.61 cm^−2^·s^−2^ (HbbH)) but only in the second patch (position 5) (Table **4**). Large fluctuations among treatments were recorded for τ_R_, showing differences with the combination of shoot density, number of patches and box position in both scenarios (Table **2**). In the biological scenario, τ_R_ values in the first patch (position 2) were lower than those recorded at adjacent positions (Figure **5**).

**Table 2:** Results of three-way ANOVA.

Factor	dƒ	Physical scenario				Biological scenario			
		SS	MS	F	P	SS	MS	F	P
**a) U velocity (cm·s^−1^)**									
Shoot density	1	361.21	361.22	70.19	**>0.001**	258.87	258.87	27.19	**>0.001**
Patch number	1	34.92	34.93	6.78	**0.001**	16.374	16.37	1.72	0.191
Position	4	323.45	80.86	15.71	**>0.001**	314.83	78.71	8.26	**>0.001**
Shoot density×Patch number	1	0.79	0.79	0.15	0.695	12.579	12.58	1.32	0.251
Shoot density×Position	4	274.15	68.53	13.32	**>0.001**	215.53	53.88	5.66	**>0.001**
Patch number×Position	4	16.89	4.22	0.82	0.513	70.27	17.57	1.84	0.121
Shoot×Patch number×Position	4	5.23	1.30	0.25	0.906	25.08	6.27	0.65	0.621
Error		1029.1	5.14			1903.70	9.51		
**b) TKE (cm^−2^·s^−2^)**									
Shoot density	1	233.37	233.37	9.10	**0.003**	51.97	51.97	0.74	0.390
Patch number	1	122.47	122.47	4.77	**0.029**	0.007	0.007	0.0001	0.991
Position	4	409.31	102.33	3.99	**0.004**	384.69	96.17	1.37	0.244
Shoot density×Patch number	1	105.96	105.96	4.13	**0.04**	122.22	122.27	1.74	0.187
Shoot density×Position	4	538.63	134.65	5.25	**0.0004**	723.48	180.87	2.58	**0.038**
Patch number×Position	4	298.57	74.64	2.91	**0.022**	248.17	62.04	0.88	0.473
Shoot×Patch number×Position	4	202.17	50.54	1.97	0.10	262.55	65.63	0.93	0.443
Error		5125.68				14010.12	70.05		
**c) Reynolds stress (Pa)**									
Shoot density	1	0.215	0.215	4.671	**0.03**	0.118	0.118	4.470	**0.035**
Patch number	1	0.005	0.005	0.114	0.735	0.015	0.015	0.582	0.446
Position	4	0.238	0.059	1.289	0.275	1.235	0.308	11.656	**>0.001**
Shoot density×Patch number	1	0.004	0.004	0.104	0.746	0.024	0.024	0.910	0.341
Shoot density×Position	4	0.121	0.030	0.657	0.622	0.213	0.053	2.012	0.09
Patch number×Position	4	0.052	0.013	0.285	0.887	0.421	0.105	3.977	**0.004**
Shoot×Patch number×Position	4	0.591	0.147	3.200	**0.01**	0.355	0.088	3.358	**0.01**
Error		9.242	0.046			5.295	0.026		

The hydrodynamic variables were tested with the factors “shoot density, “number of patches” and “position” in both the physical and biological scenarios. Significant differences are shown in bold.

**Table 3:** Flow velocity in the different scenarios (Phy = physical; Bio = biological), treatments and positions along the racetrack flume.

		Velocity (cm·s−^1^)				
Scenario	Treatment	Position 1	Position 2	Position 3	Position 4	Position 5
Phy	bbbb	15.82±0.37	16.36±0.21	16.38±0.30	16.46±0.22	16.06±0.56
	Lbbb	16.10±0.38	14.65±0.81	14.35±0.81	15.67±0.70	13.99±0.61
	LbbL	15.27±0.14	14.15±0.62	14.15±0.50	13.25±0.59	12.89±0.69
	Hbbb	15.69±0.17	14.64±0.71	11.87±1.97	9.33±0.86	8.41±0.26
	HbbH	14.57±0.20	14.29±0.82	12.11+1.65	8.38±0.81	6.85±0.93
Bio	bbbb	16.26±0.30	16.37±0.20	15.90±0.18	16.55±0.11	16.61±0.20
	Lbbb	14.43±0.27	13.59±0.68	12.59±1.19	14.04±0.75	13.93±0.79
	LbbL	15.46±0.13	14.13±0.71	13.69±0.63	12.98±0.46	12.71±0.52
	Hbbb	15.28±0.14	14.62±0.97	8.52±2.03	10.16±1.55	10.65±1.32
	HbbH	14.85±0.23	13.65±1.05	8.73±1.74	10.26±1.80	6.10±1.95

Data show the average for the canopy height (18 cm) ± SD. L, low shoot density; H, high shoot density and b, bare sediment.

**Table 4:** TKE (cm^−2^·s^−2^) and Reynolds stress (τ_R_, Pa) values in the different scenarios (Phy = physical; Bio = biological), treatments and positions along the racetrack flume (experimental details in Figure 1).

		Position 1		Position 2		Position 3		Position 4		Position 5	
Scenario	Treatment	TKE	τ_R_	TKE	τ_R_	TKE	τ_R_	TKE	τ_R_	TKE	τ_R_
Phy	bbbb	0.25±0.076	0.15±0.036	0.29±0.06	0.15±0.02	0.28±0.10	0.14±0.039	0.28±0.12	0.13±0.04	0.38±0.17	0.14±0.05
	Lbbb	0.14±0.07	0.09±0.04	2.98±1.65	0.24±0.09	0.54+0.07	0.11±0.02	0.92±0.10	0.09±0.02	1.05±0.05	0.09±0.01
	LbbL	0.03±0.01	0.04±0.01	4.55±4.11	0.09±0.04	0.55±0.21	0.14±0.05	0.45±0.11	0.17±0.02	0.61±0.03	0.17±0.02
	Hbbb	0.15±0.07	0.09±0.03	4.67±1.78	0.12±0.04	0.88±0.7	0.29±0.16	2.11±0.83	0.25±0.12	1.54±0.66	0.27±0.13
	HbbH	0.10±0.04	0.08±0.02	8.30±3.50	0.34±0.14	14.77±5.82	0.30±0.09	0.63±0.13	0.13±0.06	1.47 ± 0.51	0.07±0.03
Bio	bbbb	0.03±0.01	0.02±0.01	0.16±0.07	0.03±0.02	0.05±0.27	0.03±0.02	0.04±0.02	0.02±0.01	0.12±0.06	0.05±0.03
	Lbbb	0.04±0.01	0.03±0.02	11.47±11.03	0.00±0.09	2.64±1.88	0.11±0.03	0.49±0.11	0.19±0.03	0.44±0.07	0.20±0.03
	LbbL	0.047±0.01	0.03±0.01	3.84±2.98	0.07±0.05	1.39±0.80	0.10±0.02	0.46±0.06	0.17±0.01	0.62±0.12	0.19±0.02
	Hbbb	0.04±0.01	0.04±0.01	1.55±0.85	−0.42±0.07	3.93±2.33	0.03±0.90	2.75±0.56	0.33±0.07	3.95±0.65	0.55±0.098
	HbbH	0.17±0.11	0.04±0.01	3.96±2.65	0.07±0.04	2.98±1.65	0.18±0.08	1.74±0.71	0.22±0.05	12.41±6.61	0.19±0.10

Data are the average for the canopy height (18 cm) ± SD. L, low shoot density; H, high shoot density and b, bare sediment.

### Concentration and availability of resources

**Figure 6:**
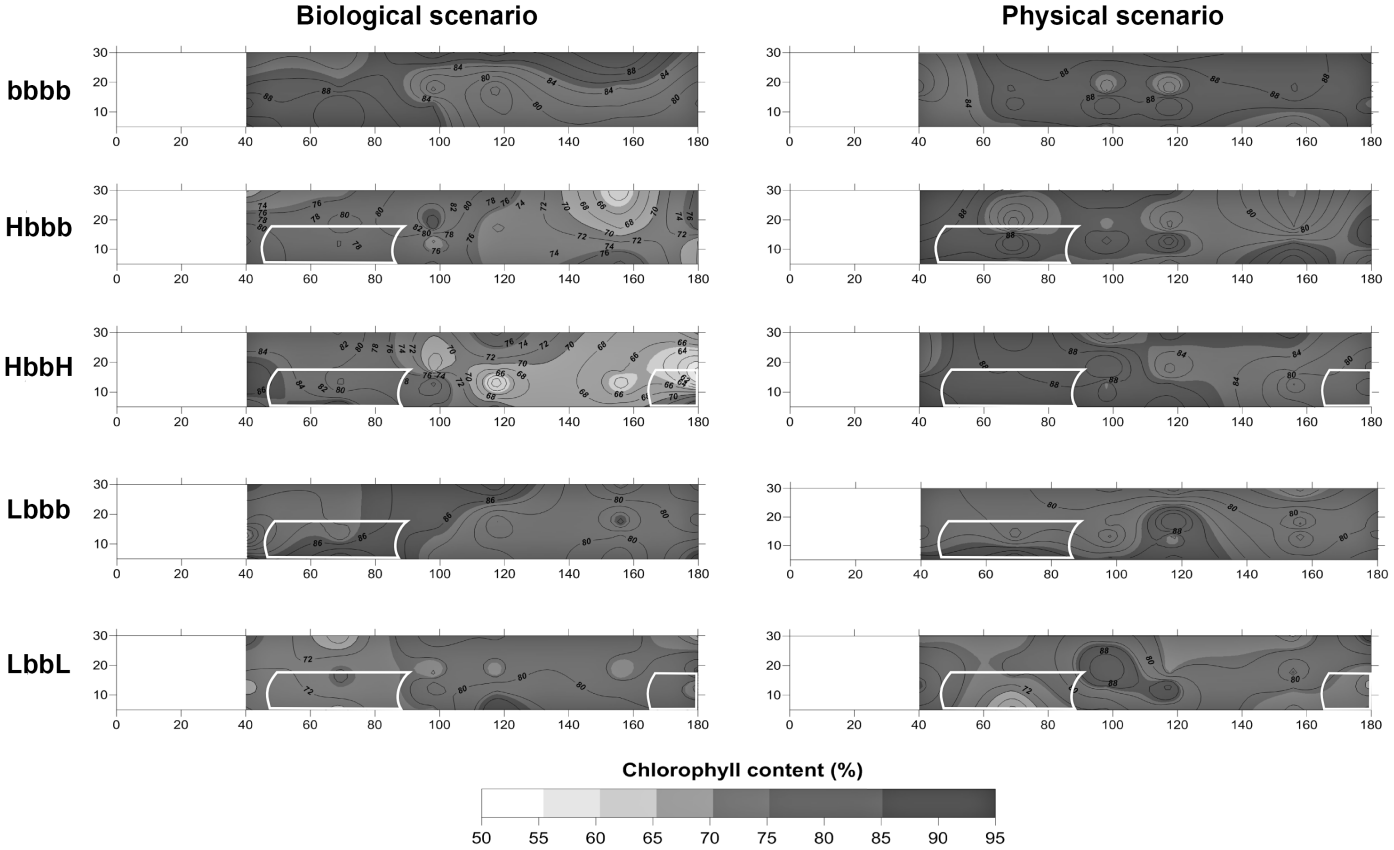
Water chlorophyll a content. Mean values (n = 3) were interpolated along the test section (x/z plane) as a percentage (%), where 100% is the initial concentration of chlorophyll *a* measured following the addition of the algae culture and 0% is the total absence of chlorophyll *a*.

In all the treatments applied in the physical scenario, the final water chlorophyll *a* concentration (Chla_fin_) was between 80–95% of the initial values, showing no noticeable differences with control treatment (bbbb). This trend varied slightly in the treatment LbbL where values decreased to 75% within the first patch (position 2). In the biological scenario, chlorophyll *a* remained between 80–95% in the control as well as in the low shoot density treatments (Lbbb and LbbL). However, a remarkable decrease (up to 50% of the initial value) was recorded in the high shoot density treatments (Hbbb and HbbH) (Figure **6**).

### Interaction between mimics and cockles

**Figure 7:**
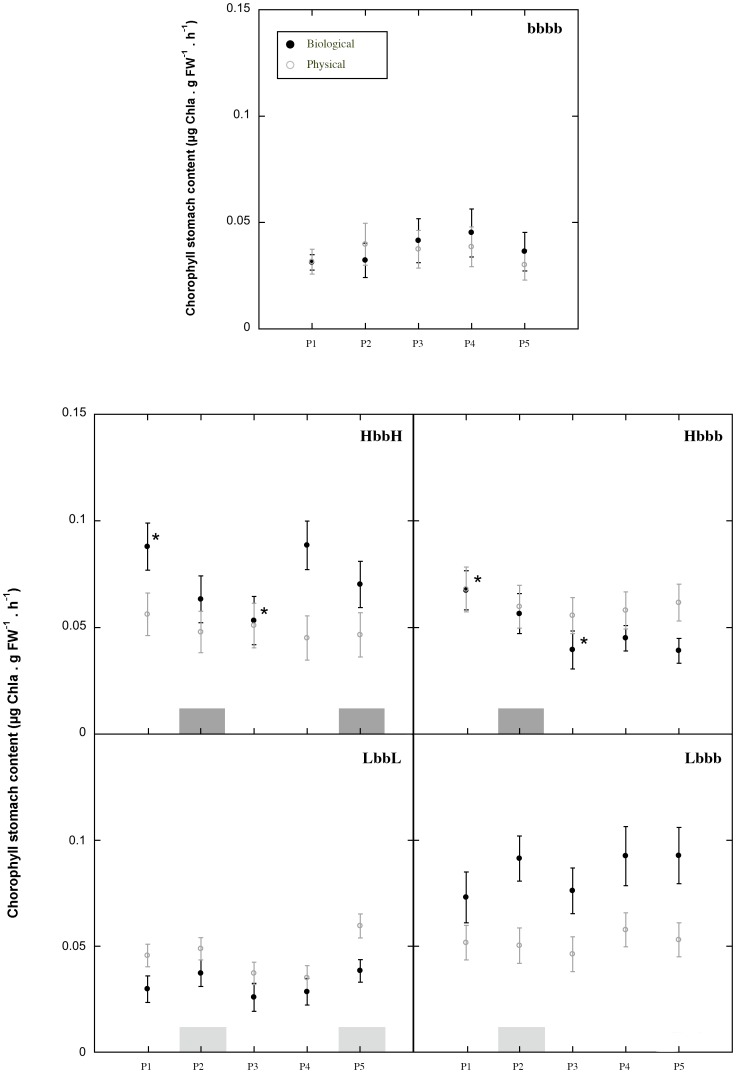
Mean chlorophyll stomach content of the cockles along the racetrack flume for different treatments and scenarios. Grey squares indicate the position of the patch (dark grey indicate high shoot density and light grey indicate low shoot density). Asterisks denote significant differences tested by the Kruskal-Wallis test (*p*-value<0.05). P (*1–5*) refers to the positions hosting the cockles along the racetrack flume.

Overall, “experimental cockles” ingested 25% more chlorophyll when occurring at high (biological scenario) than at low cockle density (physical scenario). Spatially, no significant differences in FIR were found among treatments (i.e. shoot density and number of patches) in the physical scenario, although cockles ingested more chlorophyll in the vegetated treatments than in the control ones in both scenarios (Figure **7**). Contrastingly, the chlorophyll stomach content of the individuals located either at position 1 (ahead of the forefront patch) or 3 (behind the forefront patch) of the high shoot density treatments (Hbbb and HbbH) of the biological scenario was significantly higher than that found in the adjacent positions. No significant differences among positions were found either in the control (bbbb) or in the low-density patches (Lbbb and LbbL; Figure **7**), but cockles ingested significantly more chlorophyll in treatment Lbbb of the biological scenario.

## Discussion

This work provides the first quantitative evidence that food availability to filter feeders is modulated by seagrass patch complexity (i.e. shoot density and spatial patch arrangement). Furthermore, it also shows that filter feeder density is a key factor controlling food concentration for such organisms inhabiting seagrass meadows. Our experimental design created a matrix balancing both the hydrodynamic food supply rate (controlled by the seagrass characteristics) and the food consumption rate (controlled by cockle density), thereby generating different food supply gradients. These gradients affected the food intake rate (FIR) of individuals (“experimental cockles”), depending on their specific spatial position within the flume. This was especially noticeable in the biological scenario, where cockles at position 2 (ahead of the forefront patch) exhibited the highest FIRs with high shoot density treatments (Hbbb and HbbH). This could result from variations in TKE and τ_R_ associated with the pumping activity of cockles (i.e. biomixing) observed in this work.

### Interaction between mimics and flow

In agreement with previous studies [33], [34], [45], [46], [54], our results showed the development of a turbulent patch–wake behind the forefront patch, an increase of TKE at the edges of the patch and a gradual reduction of the 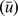 velocity within the seagrass canopy. The strongest reduction of 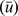 velocity was recorded within the high-density shoot patches, with highest TKE-values at the edges. In contrast, in the low-density shoot patches, the edge effect was attenuated due to the greater permeability of the seagrass canopy and the higher values of volumetric flow rate [32], [54]. A reduction in the TKE wake was observable between the Hbbb and HbbH treatments. Such effects have been previously described by Folkard [54], where the water velocity was quickly decreased between two high density patches, indicating that the wake was weakened by interaction with the second patch. When the gaps between the patches are small, there is not enough space for wake formation between them, and the hydrodynamic characteristics of a theoretically homogeneous meadow prevail. The reduction of 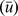 velocity associated with the presence of a second patch (in position 5) at some distance downstream from the forefront one (in position 2) indicated that both density and number of patches modulated the flow speed. The differences in TKE and τ_R_ within the forefront patch observed between physical scenario and biological scenario demonstrated how the presence of high densities of cockles influenced flow characteristics via the exhalant jets of their siphons (i.e. bio-mixing *sensu* [17], [19], [55]).

### Resource concentration balance

Even though turbulence increased at the patch edges of the high shoot density treatment (i.e. HbbH) of the physical scenario, volumetric flow rate diminished. This concentrated a high percentage of chlorophyll *a* behind the forefront patch (position 2), while this percentage lessened when the mass of water moved downstream away from this patch. The edge effect seemed to enhance trapping and sedimentation of particles in accordance with previous studies [36]. Therefore, these areas contain plenty of edible particles (i.e. phytoplankton) available for filter feeders. By contrast, in the low shoot density canopies of the physical scenario, the attenuated edge effect and the high volumetric flow rate prevented any food depletion within the patch. Surprisingly, no differences in the FIRs were found among treatments or positions within the physical scenario (i.e. spatial differences) suggesting a large availability of resources for cockles in all treatments due to (1) high resource renewal exceeding the rate of consumption and (2) the absence of intraspecific competition. Such an idea was also supported by the absence of chlorophyll *a* gradients in the water, which indicated that food availability was not limiting in the physical scenario, although an increase in the filtering efficiency of the organisms due to reduction of the flow velocity had been expected [26], [28], [38].

Even though no strong variation in water chlorophyll *a* was observed in the low shoot density treatments (Lbbb and LbbL) of the biological scenario, depletion was clearly associated with high-density patches (Hbbb and HbbH). In the low shoot density treatments—despite the higher resource consumption by the dense cockle bed—the unidirectional flow passed freely through the seagrass patch as in the control (unvegetated, bbbb) treatments, which refreshed the water across the canopy and avoided the formation of chlorophyll *a* gradients above cockles. This may explain why cockles in the low-density shoot treatments (Lbbb and LbbL) had similar FIRs, regardless of spatial location along the racetrack flume. In opposition, large variations in water chlorophyll *a* were detected in the high-density shoot treatments (Hbbb and HbbH), leading to a reduction of up to 50% of the initial chlorophyll *a* concentration behind the forefront patch. This pattern was correlated with the noticeable spatial differences found in the cockle FIR: two peaks of ingestion were observed with one ahead of (position 1) and another behind (position 3) the first patch. This agrees well with the increment in turbulence and the sharp water speed reduction observed within the canopy, where the organisms were able to capture the particles before total depletion of chlorophyll.

### Filter feeder food intake rate

Cockle FIR was higher in all seagrass treatments than in control ones (i.e. unvegetated). Present findings are in agreement with previous studies reporting positive effects on growth and survival of clams inhabiting seagrass meadows [6], [7], [10]. For the biological scenario we expected lower FIRs due the strong intraspecific competition according to the reduction of chlorophyll *a* detected in Hbbb and HbbH treatments. However, cockles surprisingly ingested 25% more chlorophyll than under the physical scenario (i.e. very low cockle density). Such an increase in FIR could be initially explained by the alteration of their foraging behavior in response to resource depletion [44], [56], [57]. However, our results suggest that biomixing could also play an important role, allowing cockles to access additional resources. Filtering (FIR) has been shown to be affected—especially at low velocity regimes—by the biomixing generated by the pumping of other bivalves [19], [55], [58]. Siphon inhalation continuously withdraws particles from the bulk water, while exhalation feathers favor vertical water mixing, thus altering the structure of the bottom boundary layer and enhancing the refreshment of resources in depleted environments [19], [44], [59], [60]. Considering that bivalves change their feeding behavior depending on particle concentration in the water column [38], [44], [56], [57], or on the population density [61], food concentration and vertical mixing promoted by the exhalant feathers might be reciprocally modulated [19], [44], [58]. Accordingly, Sobral and Widdows [38] reported that the bivalve *Ruditapes decussatus* caused a significant depletion of the phytoplankton concentration at low water velocities but maintained high filtration rates by ejecting water at different heights in the water column (i.e. biomixing), avoiding the recirculation of algal cell-depleted water in the surroundings of the intake siphons. Also, Riisgård and Larsen [58] pointed out that biomixing enhanced the flow-induced down-mixing of phytoplankton, which will benefit the turnover of the low-chlorophyll concentration water layer and could be identified as peaks in profiles of turbulent shear stress and turbulent kinetic energy.

### Ecological relevance

**Figure 8:**
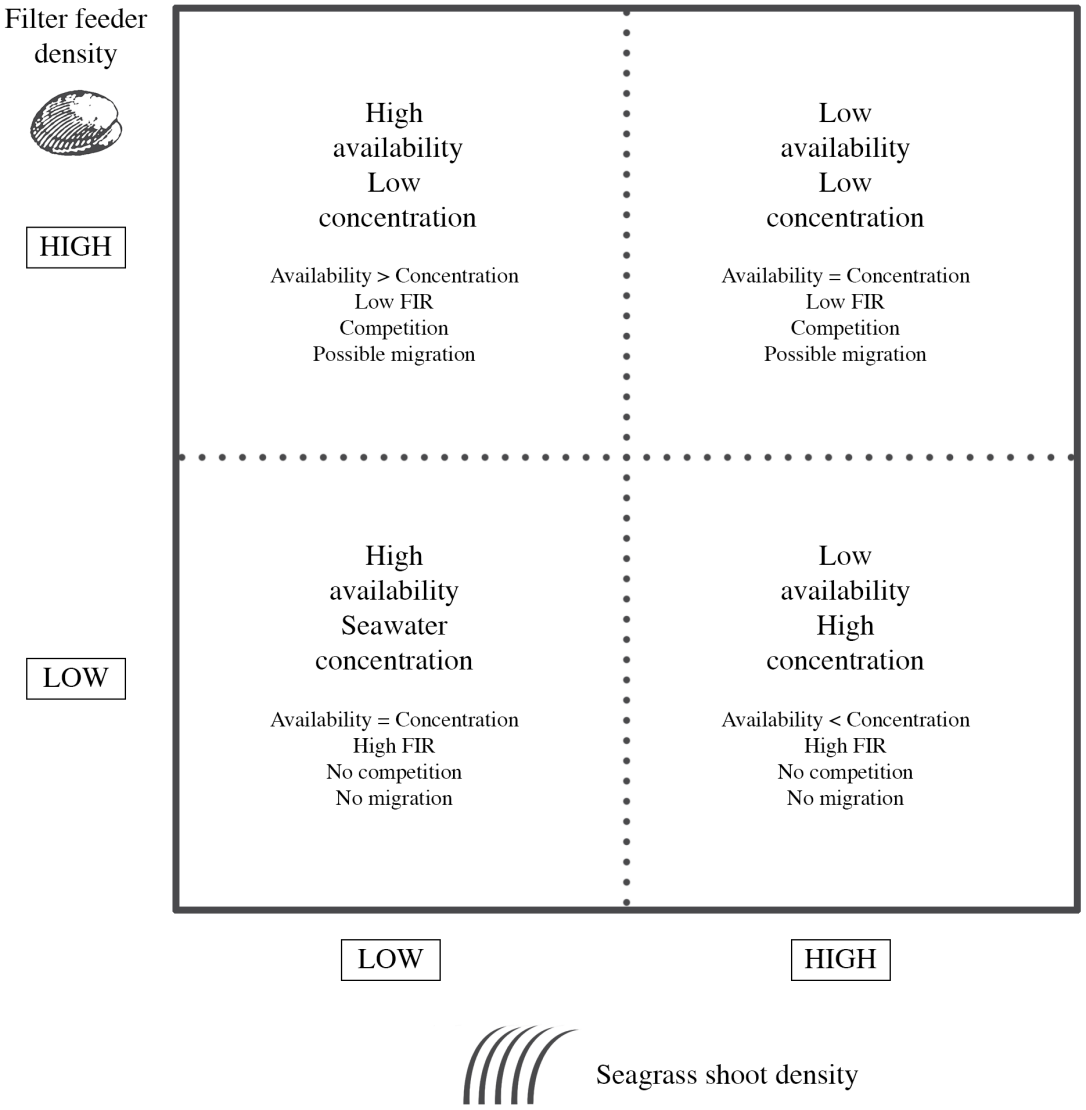
Conceptual model showing the effects of filter feeders and shoot density on resource availability and concentration. Higher shoot densities reduce resource availability (e.g. lower volumetric flow rate) but may increase resource concentration (e.g. deposition or settling). Higher density of filter feeders will reduce resource concentration (e.g. active filtration by organisms) but also may increase biomixing. Thus, the balance between availability and concentration of resources may promote changes at the community levels (e.g. migration of species depending on resources availability).

This work showed that food availability in seagrass meadows is the outcome of a complex interaction between hydrodynamic forces and habitat complexity together with intra-specific competition (consumption and bio-mixing). Our results suggest the existence of a direct correlation between plants (mimics) and bivalve density (Figure **8**): fragmented and sparse seagrass meadows could offer protection and increase food availability (higher particles flux) for filter feeders without retaining the particles within the canopy. In opposition, continuous and dense meadows enhance the concentration of resources (e.g deposition or settling) favoring food intake but will limit resources at high filter feeder densities due to the lower turnover of the water and to the depletion of resources promoted actively by organisms. Under such scenario, the activity of the filter feeders demonstrated to play also an important role, contributing to reduce resource starvation.
